# Identification of hub genes in the crosstalk between type 2 diabetic nephropathy and obesity according to bioinformatics analysis

**DOI:** 10.1080/21623945.2024.2423723

**Published:** 2024-11-11

**Authors:** Shaomin Shi, Ke Ding, Feng Chen, Mei Yang, Lihua Ni, Xiaoyan Wu

**Affiliations:** a Department of Nephrology, Zhongnan Hospital of Wuhan University, Donghu Road, Wuhan, Hubei, China; b Xiangyang Central Hospital, Affiliated Hospital of Hubei University of Arts and Science, Xiangyang, Hubei, China; c Department of General Practice, Zhongnan Hospital of Wuhan University, Donghu Road, Wuhan, Hubei, China

**Keywords:** Diabetic nephropathy, obesity, differentially expressed genes, hub genes, bioinformatics

## Abstract

Diabetic nephropathy (DN) and obesity bring a huge burden to society. Obesity plays a crucial role in the progression of type 2 DN, but the pathophysiology remains unclear. Thus, we aimed the explore the association between type 2 DN and obesity using bioinformatics method. The gene expression profiles of type 2 DN (GSE96804) and obesity (GSE94752) were downloaded from the Gene Expression Omnibus (GEO) database. The differentially expressed genes (DEGs) were screened with the thresholds defined as |log2FC| ≥1 and P<0.05. Gene Ontology (GO) and Kyoto Encyclopedia of Genes and Genomes (KEGG) enrichment analyses were performed. Subsequently, a protein-protein interaction network was constructed based on the STRING database. Hub genes were identified, and the co-expression network was constructed. Finally, the hub genes were verified in clinical samples of 24 patients by immunohistochemistry. A total of 17 common DEGs were identified. Finally, two overlapping hub genes were identified (CCL18, C1QC). C1QC has been verified in clinical specimens. Using bioinformatics methods, the present study analyzed the common DEGs and the potential pathogenic mechanisms involved in type 2 DN and obesity. C1QC was the hub gene. Further studies are needed to clarify the specific relationships among C1QC, type 2 DN and obesity.

## 1. Introduction

Diabetic nephropathy (DN) is one of the most common microvascular complications of type 2 diabetes and is the leading cause of end-stage renal disease in diabetic patient [1–3]. The pathogenesis of DN remains elusive, and multiple factors including hyperglycaemia, haemodynamic factors, inflammation, and oxidative stress, are involved [4,5].

Obesity is a growing social problem that can lead to serious consequences in various organs, such as diabetes, non-alcoholic fatty liver disease, and cardiovascular disease, which impose a heavy burden on society [6–11]. The prevalences of overweight and obesity have risen dramatically in the past several decades, and it is predicted that by 2030, approximately 57.8% of the elderly population will be overweight or obese [12].

Many previous reports have discussed obesity-related glomerulopathy [4]. In the past decade, a growing number of studies have indicated that obesity plays a crucial role in the progression of DN [12,13], leading to a higher prevalence and more rapid development of DN in individuals with obesity and type 2 diabetes [4]. As our previous study showed, obesity, especially abdominal obesity, is a risk factor for DKD, which may be explained by, among other factors, an increased inflammatory response, insulin resistance, endothelial dysfunction, fibrosis, arteriosclerosis, thrombosis, and lipotoxicity [4,8,14,15]. Moreover, increased ectopic fat deposition in the kidneys, in combination with obesity, might further promote DN [16]. However, the exact underlying mechanisms have not yet been fully clarified, such as the specific genes, proteins and signalling pathways involved.

An improved understanding of the pathophysiology of DN may enable new management and detection methods in the future. To our knowledge, although studies have separately explored the core genes of type 2 DN and obesity [17,18]. No studies have explored the association between type 2 DN and obesity via bioinformatics analyses. Therefore, based on bioinformatics technology, this paper was designed to identify the coexpressed differentially expressed genes (DEGs) associated with type 2 DN and obesity, and to analyse the possible pathways involved to provide a new direction for exploring the pathological contribution of obesity to type 2 DN. In addition, hub genes were identified by constructing a PPI network. Finally, two other public gene datasets and clinical specimens were used for verification.

## 2. Materials and methods

### 2.1. Raw data collection

The GEO (http://www.ncbi.nlm.nih.gov/geo) is a public database that contains a large number of high-throughput sequencing and microarray datasets submitted and shared by researchers worldwide. Related gene expression datasets were searched for in the GEO using ‘diabetic nephropathy OR diabetic kidney disease’ and ‘obesity’ as keywords, and with restrictions to limit the results to ‘Expression profiling by array’ and ‘samples from human beings’. The research samples used were as follows: GSE96804 (platform: GPL17586; Affymetrix Human Transcriptome Array 2.0; the original data included 41 late-stage type 2 DN patients and 20 healthy controls, and the samples were of glomeruli); and GSE94752 (platform: GPL11532; Affymetrix Human Gene 1.1 ST Array; the original data included 9 healthy lean controls, and 39 obese women, and the samples were of abdominal subcutaneous white adipose tissue).

### 2.2. Identification of the DEGs

The probe names were all converted to gene symbols after standardizing the datasets. Those without corresponding gene symbols or duplicate ones were deleted, and genes with multiple probes were averaged. The DEGs were screened by limma package of R software (version:4.2.2), with the thresholds defined as |log_2_FC| (FC: Fold Change)≥1 and *p* < 0.05. Volcano maps were drawn by ggplot2 packages of R software, and intersection of DEGs was taken by VennDiagram package.

### 2.3. Enrichment analyses of the intersection of the DEGs

To further study the functions of the DEGs, clusterProfiler package of R software (version:4.2.2) was used for gene functional enrichment and signalling pathway analysis, and the ggplot2 package was used for visualization of analysis results, based on the Gene Ontology (GO) and the Kyoto Encyclopaedia of Genes and Genes (KEGG) notes. An adjusted *p* value < 0.05 was considered to indicate significant enrichment.

### 2.4. Protein-protein interaction network construction

String 11.5 (https://cn.string-db.org/), an online search tool for retrieving the complex regulatory relationships of interacting genes, was used to construct a protein-protein interaction (PPI) network of DEGs, with combined confidence score set to be 0.4. Cytoscape is an open-source network display and analysis software for users, primarily for the analysis of large-scale protein-protein interactions, protein-DNA and genetic interactions. The PPI network was visualized by Cytoscape 3.9.1 (https://www.cytoscape.org).

### 2.5. Selection and analysis of the hub genes

The plug-in cytoHubba of Cytoscape was used to identify the hub genes. Eight common algorithms (MCC, MNC, Degree, EPC, DMNC, BottleNeck, Closeness, Radiality) were used to obtain the top 6 genes separately, and then their intersection was taken by VennDiagram package of R software.

Afterwards, based on GeneMANIA (http://www.genemania.org/), a coexpression network of these hub genes were constructed. GeneMANIA is a reliable tool that can identify associations within gene sets. Given a query list, GeneMANIA uses available genomics and proteomics data to expand into functionally similar gene.

### 2.6. Validation of the hub genes

#### 2.6.1. Validation by datasets

GSE111154 was used for validation in DN patients (platform: GPL17586; Affymetrix Human Genome U133A 2.0 Array; the original data included 4 early diabetic nephropathy patients and 4 nondiabetic controls, and the samples were of glomeruli). In addition, GSE133786 was chosen for validation in obese patients (platform: GPL571; Affymetrix Human Genome U133A 2.0 Array; the original data included 6 obese patients and 5 healthy controls, and the samples were of visceral fat tissues).

#### 2.6.2. Validation by clinical specimens

The study was approved by the Ethics Committee of Xiangyang Central Hospital, an affiliated hospital of Hubei University of Arts and Science, and was performed in accordance with the guidelines of the Declaration of Helsinki. Written informed consent was obtained from each participant (ethics batch number: XYSZXYY-LLDD-PJ-2023-104).

Six patients diagnosed with DN and six controls without DN, who underwent renal biopsy at Xiangyang Central Hospital from September 2023 to March 2024 were selected for our research, and kidney samples were collected (The duration was determined based on the sample size calculated and the frequency of kidney biopsy and bariatric surgery performed in our hospital). Moreover, six obese patients (BMI ≥28 kg/m^2^) who underwent bariatric surgery, and six lean controls (BMI <24 kg/m^2^) who underwent laparoscopic surgery at Xiangyang Central Hospital were selected, and the visceral adipose tissue (VAT) (from omental adipose tissue) and subcutaneous adipose tissue (SAT) samples were collected. The inclusion criteria for the DN group were as follows: (1) had a diagnosis of type 2 DN and (2) were aged between 18 and 80 years. The inclusion criteria for the non-DN control group were as follows: (1) type 2 diabetes patients without a DN diagnosis and (2) aged between 18 and 80 years. The inclusion criteria for the obesity group were as follows: (1) BMI ≥28 kg/m^2^ and (2) aged between 18 and 80 years. The inclusion criteria for the lean control group were as follows: (1) BMI <24 kg/m^2^ and (2) aged between 18 and 80 years. Patients with abdominal infections were excluded.

The basic parameters were collected from the participants, including medical history, age, and sex. Height and weight were also collected for patients whose abdominal subcutaneous fat and visceral fat were retained.

### 2.7. Immunohistochemisty

Kidney tissues and adipose tissues were fixed in 4% paraformaldehyde solution, dehydrated, and embedded in paraffin for sectioning into 4-mm thick sections. Immunohistochemistry for CCL18 (Abs141038; Abcam, UK) and C1QC (Ab300057; Abcam, UK) were performed on paraffin-embedded sections. Microwave oven-based antigen retrieval was performed using citric acid antigen repair buffer (pH 6.0). The sections were then incubated with 3% BSA for 30 min. Placed flat in a wet box, the sections were incubated with the primary antibody overnight at 4°C. Then, the sections were placed flat in a wet box and incubated with the secondary antibody overnight at 4°C. Positive antibody binding was visualized using a DAB peroxidase substrate kit (2005289, China). The sections were dehydrated to make them transparent and then stained with haematoxylin. The sections were sealed with neutral gum. Image-Pro Plus 6.0 (Media Cybernetics, Inc., Rockville, MD, USA) software was used to quantify the positive expression of the two hub genes.

### 2.8. Statistical analysis

To confirm the results, Stata Version 16 (Stata Corporation, College Station, TX, USA) was used for statistical analysis. Differences in variances between groups were estimated by 2-tailed Student’s t test. A *p* value < 0.05 was considered to indicate statistical significant.

The minimum sample sizes in the validation sets were calculated using PASS 15.0. In this study, samples were retained during the operation after the patients’ full informed consents, and there was no sample loss. According to the mRNA expression data in the dataset GSE96804 and the dataset GSE94752, for the validation of the expression of C1QC in type 2 DN patients compared with controls, the calculated minimum sample size was 6 in each group (Power = 0.70, α = 0.05, Group allocation was equal [N1=N2], μ1 = 6.3, μ2 = 8.3, σ1 = 0.8, σ2 = 1.5); for the validation of C1QC in obesity patients compared with controls, the calculated minimum sample size was 4 in each group (Power = 0.70, α = 0.05, Group allocation was equal [N1=N2], μ1 = 8.9, μ2 = 10.3, σ1 = 0.7 σ2 = 0.4). Since CCL18 was considered as a circulating factor which might be expressed at low levels in tissues when measured by immunohistochemistry, the sample sizes for validation were calculated according to the expression of C1QC instead of CCL18.

## 3. Results

### 3.1. Identification of DEGs in the type 2 DN and Obesity datasets

**Figure 1.**
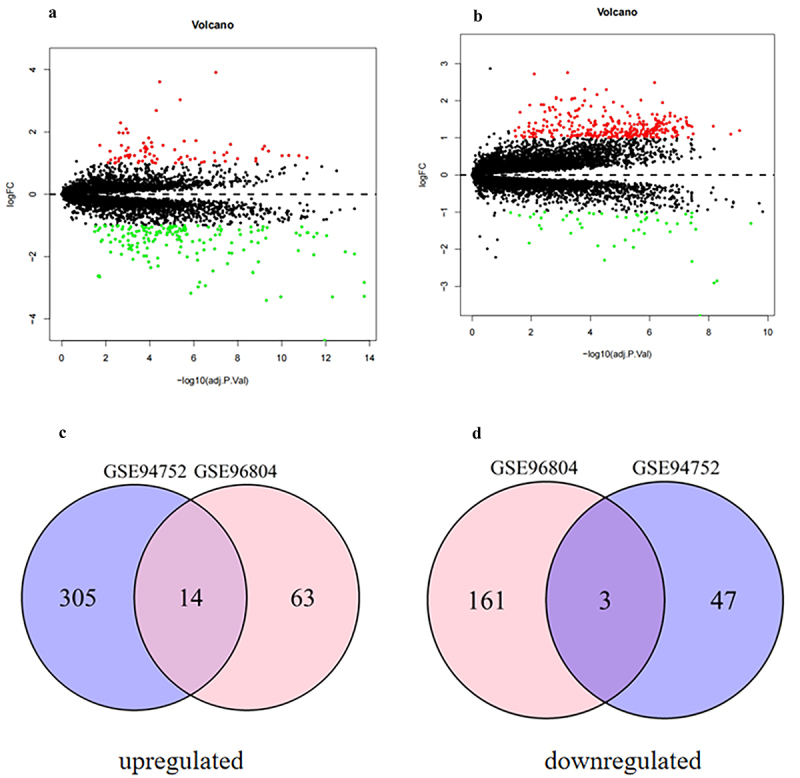
Volcano diagram and Venn diagram. (a) The volcano map of GSE96804 (DEGs in the kidneys of type 2 DN patients compared with healthy controls). (b) The volcano map of GSE94752 (DEGs in the adipose tissues of obesity patients compared with lean controls). The volcano plot shows upregulated genes (red points) and downregulated genes (green points) and genes without significance (black points). The differences threshold was set as |log2FC| >1.0 and adjusted *p* value <0.05. X-axis: Log2 fold change in gene expression. Y-axis: negative log10 p-value (or adjusted p-value). (c) The overlapping up-regulated DEGs. (d) The overlapping down-regulated DEGs. Abbreviations: DEGs indicates deferentially expressed genes.

After standardization, 241 DEGs (77 upregulated genes and 164 downregulated genes) were identified in the type 2 DN dataset (GSE96804), and 369 DEGs (319 upregulated genes and 50 downregulated genes) were identified in the obesity dataset (GSE94752) (Figure 1a,b). Finally, a total of 17 overlapping DEGs (14 upregulated genes and 3 downregulated genes) were obtained via Venn diagram analysis (Figure 1c,d). The detailed information of the overlapping DEGs is shown in Supplementary Table S1.

#### 3.1.1. Analysis of the functions of common DEGs

**Figure 2.**
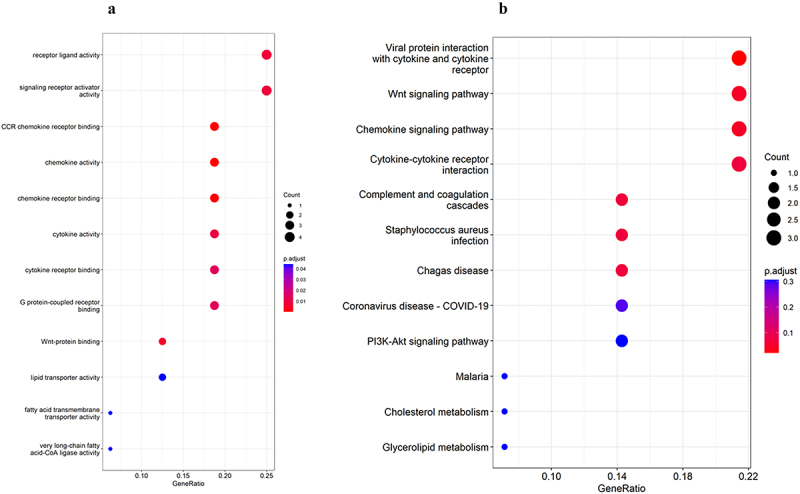
Functional enrichment of common DEGs: (a) GO enrichment analysis; (b) KEGG enrichment analysis. Abbreviations: GO indicates gene Ontology; KEGG, the Kyoto Encyclopedia of Genes and Genes; DEGs, differentially expressed genes.

To further analyse the biological functions and pathways involved in the common DEGs, we performed GO and KEGG pathway enrichment analyses. GO enrichment analysis showed that the common DEGs were mainly related to receptor ligand activity, signalling receptor activator activity, CCR chemokine receptor binding, chemokine activity, chemokine receptor binding, cytokine activity, cytokine receptor binding, G protein-coupled receptor binding, Wnt-protein binding, lipid transporter activity, fatty acid transmembrane transporter activity and very long-chain fatty acid-CoA ligase activity (Figure 2a).

In KEGG pathway enrichment analysis, the common DEGs were mainly enriched in 3 pathways: viral protein interaction with cytokine and cytokine receptor, Wnt signalling pathway, and Chemokine signalling pathway (Figure 2b).

### 3.2. PPI network construction and module analysis of common DEGs

**Figure 3.**
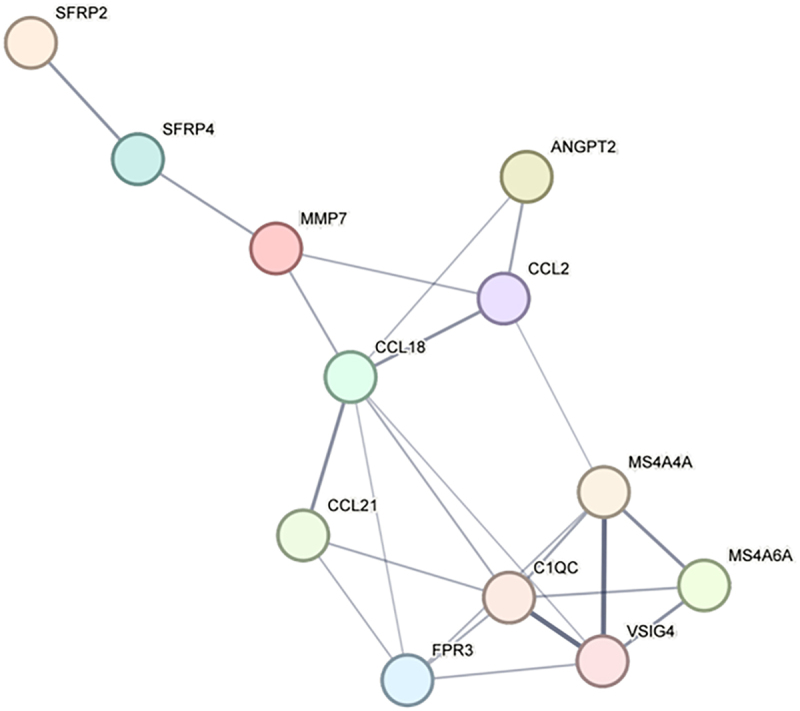
PPI network of common differentially expressed genes in type 2 DN and Obesity (constructed using STRING version 11.5). Interactions shown have a combined confidence score of ≥0.4. Nodes represent individual genes or proteins, and edges represent predicted functional associations between proteins, including direct (physical) and indirect (functional) interactions. Nodes are colored based on gene expression: red for upregulated, blue for downregulated. Edge thickness corresponds to the confidence level of the predicted interaction. Abbreviations: PPI indicates protein-protein interaction network; DEGs, differentially expressed genes.

Based on String database, the PPI network of the common DEGs was constructed using String 11.5 online software, including a total of 12 nodes and 23 interaction pairs, with the confidence score set as 0.4 (Figure 3).

#### 3.2.1. Selection and analysis of hub genes

Using the cytoHubba plug-in of Cytoscape, the top 6 genes were identified by eight algorithms (MCC, MNC, Degree, EPC, DMNC, BottleNeck, Closeness, and Radiality) (Table 1). Two overlapping hub genes, CCL18 and C1QC, were finally identified by taking the intersection of DEGs with the VennDiagram package of R software (details of the hub genes are shown in Table 2). Both were upregulated.

**Table 1.** The top 6 genes in cytoHubba.

MCC	MNC	Degree	EPC	DMNC	BottleNeck	Closeness	Radiality
C1QC	C1QC	CCL18	CCL18	MS4A4A	CCL18	CCL18	CCL18
VSIG4	VSIG4	C1QC	C1QC	CCL18	MMP7	C1QC	CCL2
FPR3	FPR3	MS4A4A	VSIG4	MS4A6A	VSIG4	MS4A4A	C1QC
CCL18	MS4A4A	VSIG4	FPR3	CCL21	CCL2	VSIG4	MMP7
MS4A4A	CCL18	FPR3	MS4A4A	VSIG4	SFRP4	FPR3	MS4A4A
MS4A6A	MS4A6A	CCL2	CCL2	FPR3	MS4A6A	CCL2	VSIG4

Abbreviations: MCC indicates maximum clique centrality; MNC, neighbourhood component centrality; EPC; edge percolated component; DMNC, maximum neighbourhood component centrality.

**Table 2.** Details of the hub genes.

No	Gene symbol	Full name	Function
1	CCL18	C-C motif chemokine 18	Chemotactic factor that attracts lymphocytes but not monocytes or granulocytes. May be involved in B-cell migration into B- cell follicles in lymph nodes. Attracts naive T-lymphocytes towards dendritic cells and activated macrophages in lymph nodes, has chemotactic activity for naive T-cells, CD4+ and CD8+ T-cells and thus may play a role in both humoral and cell-mediated immunity responses.
2	C1QC	Complement C1q subcomponent subunit C	C1q associates with the proenzymes C1r and C1s to yield C1, the first component of the serum complement system. The collagen-like regions of C1q interact with the Ca(2+)-dependent C1r(2)C1s(2) proenzyme complex, and efficient activation of C1 takes place on interaction of the globular heads of C1q with the Fc regions of IgG or IgM antibody present in immune complexes.

**Figure 4.**
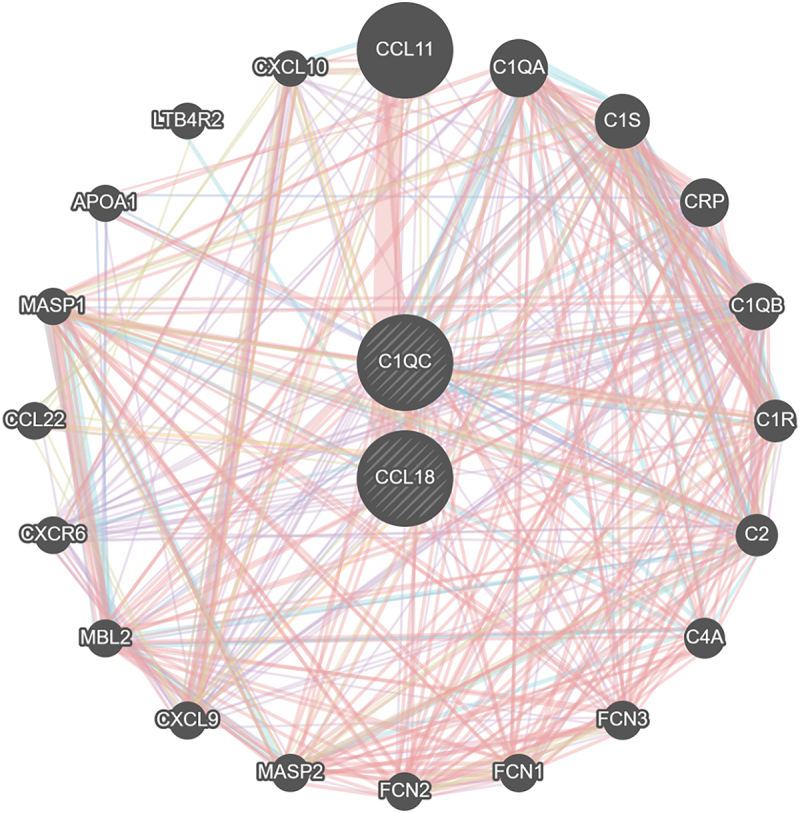
The co-expression network and functions of these hub genes.

In addition, the co-expression network and functions of these genes were analysed based on the GeneMANIA database. These genes showed the complex PPI network with the co-expression of 8.01%, physical interactions of 77.64%, colocalization of 3.63%, predicted of 5.37% and shared protein domains of 0.6% (Figure 4). These genes are mainly related to phagocytosis, humoral immune response, and complement activation.

### 3.3. Validation of the expression of the hub genes

#### 3.3.1. Validation by datasets

**Figure 5.**
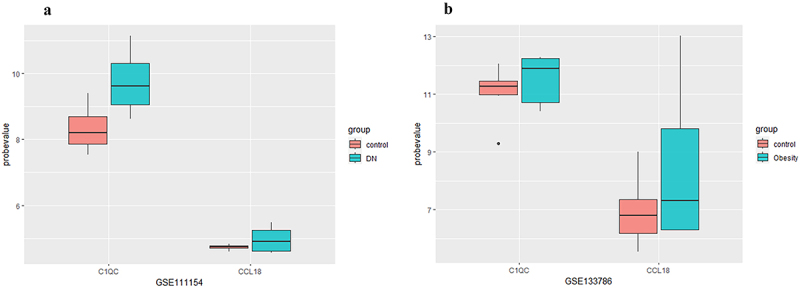
The expression of hub genes in the GSE111154 and GSE133786 datasets. Abbreviations: DN indicates diabetic nephropathy.

Both hub genes were upregulated in type 2 DN (GSE111154) and obesity (GSE133786) patients compared with the expression levels in healthy controls (Figure 5a,b), but the difference was not statistically significant (*p* > 0.05).

#### 3.3.2. Validation by clinical specimens

**Figure 6.**
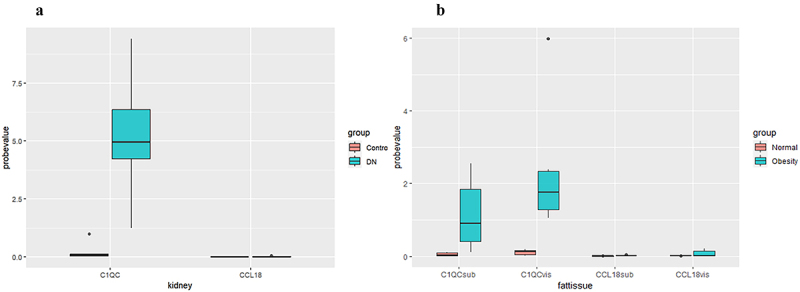
The expression of hub genes in the adipose tissues of obese patients and the kidney tissues of diabetic nephropathy patients. Abbreviations: DN, diabetic nephropathy; C1QCsub, C1QC expression in abdominal subcutaneous fat; C1QCvis, C1QC expression in visceral fat; CCL18sub, CCL18 expression in abdominal subcutaneous fat; CCL18vis, CCL18 expression in visceral fat.

**Figure 7.**
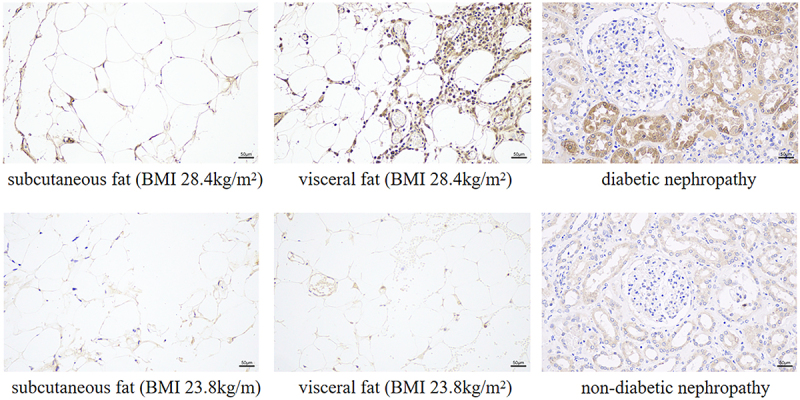
Expression of C1QC in adipose tissue and kidney tissue (immunohistochemistry, 200× field, haematoxylin staining). Abbreviations: BMI indicates body mass index.

To increase the reliability of the identification of these two hub genes, fat samples (abdominal subcutaneous fat, visceral fat) and kidney samples from 24 patients were prospectively collected in this study, and the expression of C1QC and CCL18 in the tissues of obese and diabetic nephropathy patients was verified by immunohistochemistry. Compared with that in nondiabetic nephropathy patients, the expression level of C1QC was upregulated in type 2 diabetic nephropathy patients (*p* < 0.05). Compared with that in normal controls, the expression levels of C1QC in abdominal subcutaneous fat and visceral fat were upregulated in obese patients (*p* < 0.05). CCL18 expression levels in the kidneys of type 2 diabetic nephropathy patients and in the abdominal subcutaneous fat and visceral fat of obese patients were not significantly different from those in the control groups (*p* > 0.05) (Figure 6a,b and Supplementary Table S2 and Supplementary Table S3). The immunohistochemical images of C1QC expression in abdominal subcutaneous fat, visceral fat and kidney tissue are shown in Figure 7.

## 4. Discussion

Previous studies have shown that obesity changes the function and number of different immune cells in adipose tissue [13], and adipose tissue dysfunction and inflammation might lead to diabetes and DN. Growing evidence indicates that DN is associated with obesity, but the details of this association are unclear.

In the present study, based on biological information, we explored the common DEGs in type 2 DN and obesity, and the hub genes and potential functions were analysed. A total of 14 overlapping upregulated genes and 3 overlapping downregulated genes were identified. GO analysis indicated that these genes were mainly related to receptor ligand activity, chemokine activity, cytokine activity, lipid transporter activity and other functions, while KEGG analysis revealed that they were mainly involved in viral protein interactions with cytokines and cytokine receptors, the Wnt signalling pathway, and the chemokine signalling pathway. Few studies reported the relationship between virus infection and type 2 DN or obesity. It was reported that COVID-19 infection was linked to type 2 DN, and it increased the occurrence and progression of type 2 DN [19,20]. In a cohort of COVID-19 patients (*n* = 75), it was reported that both obesity and diabetic nephropathy were linked to poor outcomes [20,21]. Meanwhile, HIV infection was reported to be related to an increased risk of abdominal obesity (OR 1.92, 1.60–2.30) and elevated low density lipid cholesterol (OR 1.76, 1.49–2.08) [22]. Another study has reported that HIV-related kidney diseases were associated with metabolic syndrome and obesity [23]. Some studies have reported other viral infections that may be associated human obesity, such as adenoviridae, herpesviridae, phages and so on [24]. Nirmal k Onteddu et al. reported that hepatitis C was associated with the hallmark sign of DN, nodular glomerulosclerosis [25]. Yang KL et al. focused on enterovirus groups, and they reported that enteroviruses and their collaboration with bacteria played an important role in the occurrence and development of both obesity and type 2 diabetes [26]. However, the specific relationships among type 2 DN, obesity and this signalling pathway (viral protein interactions with cytokines and cytokine receptors) remained unclear. In contrast, Wnt signalling pathway has been widely reported to be associated with both type 2 DN and adipogenesis, which played important roles in the inflammatory response, cell proliferation, differentiation, development, homoeostasis and self-renewal [27,28]. Moreover, chemokines, as part of pro-inflammatory factors, have also been widely considered to be closely related to type 2 DN and obesity [29,30]. All in all, the relationships among type 2 DN, obesity and the three signalling pathways needs to be further explored.

Two hub genes (CCL18 and C1QC) were ultimately identified by constructing a PPI network; these genes may both be involved in the development of type 2 DN and obesity. The hub genes are both associated with macrophages and insulin resistance and were both upregulated. Notably, to our knowledge, this is the first study to explore the correlation between type 2 DN and obesity via comprehensive bioinformatics analyses and validation in human adipose tissue and kidney tissue. CIQC has been validated in clinical specimens, although CCL18 needs to be further validated.

CCL18 is a chemotactic factor that attracts lymphocytes and it is produced by alternatively activated macrophages that contribute to inflammatory diseases [31], lung fibrosis [32], and scleroderma [33]. It was reported to synergize with high concentrations of glucose to promote the production of fibronectin in proximal tubuloepithelial cells, which may be associated with renal fibrosis in DN [34]. Several clinical studies have shown that CCL18 secreted from subcutaneous white adipose tissue is more strongly correlated with body weight, waist circumference and insulin resistance than are classic cytokines such as TNF-α and IL-6 [35].

C1q associates with the proenzymes C1r and C1s to yield C1, the first component of the serum complement system. C1QC is a C1q/TNF-related protein that is reportedly associated with adipose tissue dysfunction and the infiltration of immune cells, especially macrophages, which may contribute to insulin resistance and the development of diabetes [13,36]. Recently, it was reported to be a potential hub gene associated with the progression of obesity-induced diabetes or insulin resistance [13] and may promote DN. Moreover, previous studies have shown that renal activation of the complement system, manifested as increased C1qa, C1qb, and C1qc, contributes to renal injury in DN patients [37].

In the external dataset validation, compared with those in the normal control group, the expression levels of C1QC and CCL18 in the kidneys of type 2 diabetic nephropathy patients and in the subcutaneous or visceral fat of obese patients were upregulated, but the differences were not statistically significant. According to the results of the clinical specimen verification, immunohistochemistry showed that C1QC expression was upregulated in the kidneys of diabetic nephropathy patients (mainly in the renal tubules) and in the abdominal subcutaneous fat and visceral fat of obese patients (*p* < 0.05). However, the expression levels of CCL18 in the kidneys of diabetic nephropathy patients and in the abdominal subcutaneous fat and visceral fat of obese patients were not significantly different from those in the control groups. To validate the results in the clinical specimens, mRNA expression was measured by microarray expression profiling in the external datasets, while the tissue protein content was measured by immunohistochemistry. The results for C1QC were consistent with those expected, suggesting that it may be a target for the association between obesity and diabetic nephropathy. However, CCL18 is a chemokine that is expressed at very low levels in tissues and may be a more suitable circulating marker. Therefore, further verification of the core gene CCL18 requires the use of PCR to measure its mRNA expression or to directly measure its peripheral blood level.

Taken together, our findings indicate that C1QC and CCL18, as intermediaries between DN and obesity, may play major roles in the development of type 2 DN and obesity and may constitute the pathological mechanism by which obesity contributes to DN. Although the exact mechanisms linking obesity to DN are not well understood, the present study may pave the way for further research and provide us with a new direction to investigate the connection between obesity-induced inflammation and type 2 DN, although the exact roles and clinical value of CCL18 and C1QC remain to be defined. Current therapies for treating DN fail to stop the disease from progressing to end-stage renal disease. In the future, targeting obesity with novel drugs could constitute an additional alternative the treatment for DN.

This study has several limitations. First, the sample sizes were not large enough. Second, this was a microarray data analysis, and C1QC has been validated clinically, while CCL18 needs to be further validated. Thirdly, the results in the present study were limited to ‘Expression profiling by array’, and using a broader range of tools could provide a more comprehensive result, such as high throughput sequencing and RT-qPCR. Fourthly, potential confounding factors were not controlled in the analysis, such as other comorbidities: hypertension, cardiovascular disease, and non-alcoholic fatty liver disease, which are common in both obesity and diabetes and could affect gene expression profiles. Fifthly, the sample sizes for validation were small, and the power was set to 0.7 when calculating the minimum sample sizes, which may limit the generalizability of the findings. Selection bias in the clinical specimens might exist, as they were collected from a single hospital, and including samples from multiple centres should be considered in the future.

Thus, further studies need to consider stratifying participants by the presence of the related comorbidities or using multivariate analysis to adjust for their potential impact, and to rigorously validate and explore the signalling pathways involving the hub genes to achieve a deeper understanding of the link between type 2 DN and obesity.

## 5. Conclusions

In summary, using bioinformatics methods, the present study analysed the common DEGs and the potential pathogenic mechanism involved in the connection between type 2 DN and obesity. Two hub genes (CCL18 and C1QC) associated with both obesity and DN were ultimately identified by constructing a PPI network. CIQC has been validated clinically, but CCL18 needs to be further validated. The present study might pave the way for further research and provide us with a new direction to investigate the connection between obesity-induced inflammation and type 2 DN. Based on these results, further fundamental and clinical studies are needed to clarify the link among C1QC, type 2 DN and obesity and the detailed pathogenic mechanisms involved.

## Supplementary Material

Supplemental Material

## Data Availability

The data that support the findings of this study are mainly from the publicly available datasets (GEO, https://www.ncbi.nlm.nih.gov/geo/query/acc.cgi?acc=GSE96804 and https://www.ncbi.nlm.nih.gov/geo/query/acc.cgi?acc=GSE94752). Other relevant raw data is available at https://doi.org/10.5281/zenodo.13819501.
