# Supplementary material for: Identification of hub genes in the crosstalk between type 2 diabetic nephropathy and obesity according to bioinformatics analysis
Source: Adipocyte. 2024 Nov 11;13(1):2423723. doi: 10.1080/21623945.2024.2423723 (PMC11556279; doi:10.1080/21623945.2024.2423723)
Supplement: Supplemental Material [file KADI_A_2423723_SM3975.zip › Supplementary Table 1.doc]

**Supplementary Tabel 1** The detailed information of the overlapping DEGs

| **ID** | logFC | entrezID | Details |
| --- | --- | --- | --- |
| **ANGPT2** | 1.042 | 285 | **Angiopoietin-2**; Binds to TEK/TIE2, competing for the ANGPT1 binding site, and modulating ANGPT1 signaling. Can induce tyrosine phosphorylation of TEK/TIE2 in the absence of ANGPT1. In the absence of angiogenic inducers, such as VEGF, ANGPT2-mediated loosening of cell-matrix contacts may induce endothelial cell apoptosis with consequent vascular regression. In concert with VEGF, it may facilitate endothelial cell migration and proliferation, thus serving as a permissive angiogenic signal. TEK - Angiopoietin-1 receptor; Tyrosine-protein kinase that acts as cell-surface receptor for ANGPT1, ANGPT2 and ANGPT4 and regulates angiogenesis, endothelial cell survival, proliferation, migration, adhesion and cell spreading, reorganization of the actin cytoskeleton, but also maintenance of vascular quiescence. Has anti-inflammatory effects by preventing the leakage of proinflammatory plasma proteins and leukocytes from blood vessels. Required for normal angiogenesis and heart development during embryogenesis. Required for post-natal hematopoiesis. |
| **SFRP2** | 2.4705 | 6423 | **Secreted frizzled-related protein 2**; Soluble frizzled-related proteins (sFRPS) function as modulators of Wnt signaling through direct interaction with Wnts. They have a role in regulating cell growth and differentiation in specific cell types. SFRP2 may be important for eye retinal development and for myogenesis. |
| **CCL21** | 2.255879436 | 6366 | **CCL21 - C-C motif chemokine 21**; Inhibits hemopoiesis and stimulates chemotaxis. Chemotactic in vitro for thymocytes and activated T-cells, but not for B-cells, macrophages, or neutrophils. Shows preferential activity towards naive T-cells. May play a role in mediating homing of lymphocytes to secondary lymphoid organs. Binds to atypical chemokine receptor ACKR4 and mediates the recruitment of beta-arrestin (ARRB1/2) to ACKR4. |
| **FPR3** | 1.633211444 | 2359 | **N-formyl peptide receptor 3**; Low affinity receptor for N-formyl-methionyl peptides, which are powerful neutrophils chemotactic factors. Binding of FMLP to the receptor causes activation of neutrophils. This response is mediated via a G-protein that activates a phosphatidylinositol-calcium second messenger system. |
| **TNC** | 1.707877999 | 3371 | **TNC - Tenascin**; Extracellular matrix protein implicated in guidance of migrating neurons as well as axons during development, synaptic plasticity as well as neuronal regeneration. Promotes neurite outgrowth from cortical neurons grown on a monolayer of astrocytes. Ligand for integrins alpha-8/beta-1, alpha-9/beta-1, alpha-V/beta-3 and alpha- V/beta-6. In tumors, stimulates angiogenesis by elongation, migration and sprouting of endothelial cells. |
| **VSIG4** | 1.376841864 | 11326 | **V-set and immunoglobulin domain-containing protein 4**; Phagocytic receptor, strong negative regulator of T-cell proliferation and IL2 production. Potent inhibitor of the alternative complement pathway convertases. |
| **CCL2** | 1.657881741 | 6347 | **C-C motif chemokine 2**; Acts as a ligand for C-C chemokine receptor CCR2. Signals through binding and activation of CCR2 and induces a strong chemotactic response and mobilization of intracellular calcium ions. Exhibits a chemotactic activity for monocytes and basophils but not neutrophils or eosinophils. May be involved in the recruitment of monocytes into the arterial wall during the disease process of atherosclerosis. |
| **MS4A6A** | 1.279654288 | 64231 | **Membrane-spanning 4-domains subfamily A member 6A**; May be involved in signal transduction as a component of a multimeric receptor complex; Belongs to the MS4A family. |
| **C1QC** | 1.447761892 | 714 | **Complement C1q subcomponent subunit C**; C1q associates with the proenzymes C1r and C1s to yield C1, the first component of the serum complement system. The collagen-like regions of C1q interact with the Ca(2+)-dependent C1r(2)C1s(2) proenzyme complex, and efficient activation of C1 takes place on interaction of the globular heads of C1q with the Fc regions of IgG or IgM antibody present in immune complexes. |
| **MMP7** | 2.053558623 | 4316 | **Matrilysin**; Degrades casein, gelatins of types I, III, IV, and V, and fibronectin. Activates procollagenase. TIMP1 - Metalloproteinase inhibitor 1; Metalloproteinase inhibitor that functions by forming one to one complexes with target metalloproteinases, such as collagenases, and irreversibly inactivates them by binding to their catalytic zinc cofactor. Acts on MMP1, MMP2, MMP3, MMP7, MMP8, MMP9, MMP10, MMP11, MMP12, MMP13 and MMP16. Does not act on MMP14. Also functions as a growth factor that regulates cell differentiation, migration and cell death and activates cellular signaling cascades via CD63 and ITGB1. Plays a role in integrin signaling. |
| **CCL18** | 2.233254151 | 6362 | **C-C motif chemokine 18**; Chemotactic factor that attracts lymphocytes but not monocytes or granulocytes. May be involved in B-cell migration into B- cell follicles in lymph nodes. Attracts naive T-lymphocytes toward dendritic cells and activated macrophages in lymph nodes, has chemotactic activity for naive T-cells, CD4+ and CD8+ T-cells and thus may play a role in both humoral and cell-mediated immunity responses. |
| **SFRP4** | 1.139701362 | 6424 | **Secreted frizzled-related protein 4**; Soluble frizzled-related proteins (sFRPS) function as modulators of Wnt signaling through direct interaction with Wnts. They have a role in regulating cell growth and differentiation in specific cell types (By similarity). SFRP4 plays a role in bone morphogenesis. May also act as a regulator of adult uterine morphology and function. May also increase apoptosis during ovulation possibly through modulation of FZ1/FZ4/WNT4 signaling (By similarity). Has phosphaturic effects by specifically inhibiting sodium-dependent phosphate uptake. |
| **IGK** | 1.47981014 | 50802 | Sorry, STRING found no proteins by this name in Homo sapiens. |
| **MS4A4A** | 1.167396471 | 51338 | **Membrane-spanning 4-domains subfamily A member 4A**; May be involved in signal transduction as a component of a multimeric receptor complex. |
| **GPAT3** | -1.056057284 | 84803 | **Glycerol-3-phosphate acyltransferase 3**; Converts glycerol-3-phosphate to 1-acyl-sn-glycerol-3- phosphate (lysophosphatidic acid or LPA) by incorporating an acyl moiety at the sn-1 position of the glycerol backbone. Also converts LPA into 1,2-diacyl-sn-glycerol-3-phosphate (phosphatidic acid or PA) by incorporating an acyl moiety at the sn-2 position of the glycerol backbone ; Belongs to the 1-acyl-sn-glycerol-3-phosphate acyltransferase family. |
| **CETP** | -1.053547827 | 1071 | **Glycerol-3-phosphate acyltransferase 3**; Converts glycerol-3-phosphate to 1-acyl-sn-glycerol-3- phosphate (lysophosphatidic acid or LPA) by incorporating an acyl moiety at the sn-1 position of the glycerol backbone. Also converts LPA into 1,2-diacyl-sn-glycerol-3-phosphate (phosphatidic acid or PA) by incorporating an acyl moiety at the sn-2 position of the glycerol backbone ; Belongs to the 1-acyl-sn-glycerol-3-phosphate acyltransferase family. |
| **SLC27A2** | -1.995849839 | 11001 | **Very long-chain acyl-CoA synthetase**; Acyl CoA synthetase that activates long-chain and very long- chain fatty acids (VLCFAs) by catalyzing the formation of fatty acyl- CoA. Can also activate branched-chain fatty acids such as phytanic acid and pristanic acid. Does not activate C24 bile acids, cholate and chenodeoxycholate. In vitro, activates 3-alpha,7- alpha,12-alpha-trihydroxy-5-beta-cholestanate (THCA), the C27 precursor of cholic acid deriving from the de novo synthesis from cholesterol. Exhibits long-chain fatty acids (LCFA) transport activity and plays an important role in hepati [...] |
