# Supplementary material for: Identification of hub genes in the crosstalk between type 2 diabetic nephropathy and obesity according to bioinformatics analysis
Source: Adipocyte. 2024 Nov 11;13(1):2423723. doi: 10.1080/21623945.2024.2423723 (PMC11556279; doi:10.1080/21623945.2024.2423723)
Supplement: Supplemental Material [file KADI_A_2423723_SM3975.zip › Supplementary Table 2.doc]

**Supplementary Table 2** The expression of CCL18 and C1QC in diabetic nephropathy patients and non-diabetic nephropathy patients (immunohistochemistry)

| Number | Gender | Age | Group | C1QC | CCL18 |
| --- | --- | --- | --- | --- | --- |
| 1 | F | 47 | DN | 5.103057 | 0.003735 |
| 2 | F | 57 | DN | 6.782458 | 0 |
| 3 | M | 52 | DN | 1.241063 | 0.0540735 |
| 4 | F | 67 | Control | 0.0434007 | 0.0018783 |
| 5 | M | 56 | Control | 0.0330992 | 0.0036239 |
| 6 | F | 69 | Control | 0.0297593 | 0.0002319 |
| 7 | F | 57 | DN | 9.415666 | 0.0001035 |
| 8 | F | 57 | DN | 4.807111 | 0.0001434 |
| 9 | M | 67 | DN | 4.035669 | 0.0154789 |
| 10 | F | 61 | Control | 0.986223 | 0 |
| 11 | F | 63 | Control | 0.1262151 | 0 |
| 12 | M | 57 | Control | 0.092935 | 0.0008434 |

Abbreviations: F indicated female; M, male;DN, diabetic nephropathy; C1QC, C1QC expression in kidney; CCL18 expression in kidney.
