# Supplementary material for: Identification of hub genes in the crosstalk between type 2 diabetic nephropathy and obesity according to bioinformatics analysis
Source: Adipocyte. 2024 Nov 11;13(1):2423723. doi: 10.1080/21623945.2024.2423723 (PMC11556279; doi:10.1080/21623945.2024.2423723)
Supplement: Supplemental Material [file KADI_A_2423723_SM3975.zip › Supplementary Table 3.doc]

**Supplementary Table 3** The expression of CCL18 and C1QC in abdominal subcutaneous fat tissues and visceral fat tissues of obese patients (immunohistochemistry)

| N | H/W/BMI | Group | Gen/Age | C1QCsub | C1QCvis | CCL18sub | CCL18vis |
| --- | --- | --- | --- | --- | --- | --- | --- |
| 1 | 158/76/30.4 | Obesity | F/27 | 0.252936 | 1.322029 | 0.0037865 | 0.002805 |
| 2 | 153/66/28.2 | Obesity | F/57 | 2.131921 | 2.190998 | 0 | 0.207955 |
| 3 | 171/70/23.9 | Normal | M/52 | 0.0378319 | 0.1970698 | 0.000328 | 0.0003575 |
| 4 | 148/62/28.3 | Obesity | F/67 | 0.1129856 | 2.387615 | 0.0342539 | 0.0092922 |
| 5 | 173/70/23/4 | Normal | M/76 | 0.1104558 | 0.1221366 | 0.0012671 | 0.0109462 |
| 6 | 15855/22 | Normal | F/73 | 0.0995138 | 0.1677227 | 0.0004128 | 0.0001061 |
| 7 | 170/85/29.4 | Obesity | M/64 | 0.9400232 | 1.260727 | 0 | 0 |
| 8 | 150/66/29.3 | Obesity | F/46 | 0.8590115 | 1.051423 | 0.001283 | 0.0038459 |
| 9 | 173/85/28.4 | Obesity | M/37 | 2.543647 | 5.987845 | 0.0065722 | 0.1751728 |
| 10 | 164/54/20.1 | Normal | M/69 | 0.0164863 | 0.1155851 | 0.0000875 | 0.0051667 |
| 11 | 159/55/21.8 | Normal | F/61 | 0.0129328 | 0.0024661 | 0 | 0 |
| 12 | 145/50/23.8 | Normal | F/79 | 0.0160551 | 0.0146348 | 0 | 0.001258 |

Abbreviations: N indicates number; H, height; W, weight; Gen, gender; C1QCsub, C1QC expression in abdominal subcutaneous fat; C1QCvis, C1QC expression in visceral fat; CCL18sub, CCL18 expression in abdominal subcutaneous fat; CCL18vis, CCL18 expression in visceral fat.
